# Comprehensive Analysis of Human Cytomegalovirus- and HIV-Mediated Plasma Membrane Remodeling in Macrophages

**DOI:** 10.1128/mBio.01770-21

**Published:** 2021-08-17

**Authors:** Ramona Businger, Saima Kivimäki, Stefan Simeonov, Georgios Vavouras Syrigos, Justus Pohlmann, Michael Bolz, Patrick Müller, Marius C. Codrea, Corinna Templin, Martin Messerle, Klaus Hamprecht, Tilman E. Schäffer, Sven Nahnsen, Michael Schindler

**Affiliations:** a Institute for Medical Virology and Epidemiology of Viral Diseases, University Hospital Tübingen, Tübingen, Germany; b Institute of Applied Physics, Eberhard-Karls-University, Tübingen, Germany; c Quantitative Biology Center, Eberhard-Karls-University, Tübingen, Germany; d Institute for Virology, Hannover Medical School, Hannover, Germany; Max Planck Institute for Infection Biology

**Keywords:** HIV, antiviral immune response, human cytomegalovirus, immune receptor, viral immune evasion

## Abstract

The plasma membrane (PM) must be overcome by viruses during entry and release. Furthermore, the PM represents the cellular communication compartment and the immune system interface. Hence, viruses have evolved sophisticated strategies to remodel the PM, for instance to avoid immune sensing and clearance of infected cells. We performed a comprehensive analysis of cell surface dysregulation by two human-pathogenic viruses, human cytomegalovirus (HCMV) and human immunodeficiency virus type 1 (HIV-1), in primary macrophages, which are classical antigen-presenting cells and orchestrators of the immune system. Scanning ion conductance microscopy revealed a loss of roughness and an overall smooth phenotype of HCMV-infected macrophages, in contrast to HIV-1 infection. This phenotype was also evident on the molecular level. When we screened for cell surface receptors modulated by HCMV, 42 of 332 receptors tested were up- or downregulated, whereas HIV-1 affected only 7 receptors. In particular CD164, CD84, and CD180 were targeted by HCMV. Mechanistically, HCMV induced transcriptional silencing of these receptors in an interferon (IFN)-independent manner, and expression was reduced not only by lab-adapted HCMV but also by clinical HCMV isolates. Altogether, our plasma membrane profiling of human macrophages provides clues to understand how viruses evade the immune system and identified novel cell surface receptors targeted by HCMV.

## INTRODUCTION

A highly organized compartment that cells use to communicate with each other and with the immune system is the plasma membrane (PM) (1, 2). It contains a repertoire of receptors that can be specific for certain cell types, and therefore, receptor levels at the PM are used to phenotype cells. For instance, monocytes are marked by CD14 but do not express the T cell receptor CD3 (3, 4). Cell surface molecules have a large variety of functions and participate in the induction of intracellular signaling cascades, paracrine and autocrine cellular communication, and orchestration of the innate and adaptive immune response (4). Furthermore, the PM is a physical barrier that protects the cytoplasm from the extracellular space and regulates influx and efflux of ions and metabolites (2).

Viruses are obligatory intracellular parasites that need to overcome the PM to establish infection of the host cell (5). At late steps of the viral replication cycle, newly produced viral particles, which are released from the host cell, have to pass this barrier a second time. Alternatively, viral particles directly assemble and bud from the PM (6). Hence, PM-residing receptors are incorporated into the viral membrane.

Considering the eminent role of the PM in immune signaling and the various steps of viral replication, it seems obvious that viruses have evolved ample mechanisms to reorganize and dysregulate this compartment (7–9). In order to understand how viruses evade the immune system and subvert cellular communication, we aimed to phenotype the PM of virally infected cells at single-cell resolution. As a model, we chose primary human macrophages, which are professional antigen-presenting cells. Furthermore, macrophages are highly relevant *in vivo* targets of two pathogenic viruses that cause chronic and latent infections, HIV-1 and human cytomegalovirus (HCMV) (10, 11).

HIV-1, the causative agent of AIDS, infects CD4-positive cells, mainly T cells but also macrophages. Macrophages represent an important viral reservoir, contribute to early dissemination of HIV-1 into various organs, and play a major role in AIDS pathogenesis (10). HIV-1 is assembled and released from the PM of CD4^+^ T cells, whereas in macrophages, the virus is stored in intracellular virus-containing compartments (VCCs) (6). These might represent an immune privileged niche, as they shield HIV-1 from neutralization by antibodies and transfer the virus to adjacent T cells upon cell-to-cell interaction (12–14).

HCMV causes latent infection in humans and can induce life-threatening diseases in newborns or immunosuppressed patients (15). HCMV has a broad cell tropism and infects epithelial cells, fibroblasts, and endothelial cells as well as monocytes and macrophages (15–17). HCMV, similar to HIV-1, is a highly immunomodulatory virus and has evolved sophisticated strategies to evade the antiviral immune response (18, 19). For instance, HIV-1 and HCMV encode viral proteins that reduce the surface expression of major histocompatibility complex type I (MHC-I) to escape lysis by cytotoxic T cells (20, 21). Other examples are HCMV pUL16 and pUL141, which downregulate the natural killer cell (NK) receptors MIC-B and CD155, respectively (22, 23), and HIV-1 Nef and Vpu, which have similar activities (24–27). Apart from these specific examples, several studies assessed the regulation of single cell surface receptors by HIV-1 and HCMV, and elegant studies from the Lehner lab used unbiased proteomic profiling of the PM to uncover the complex phenotype of cell surface dysregulation in an HIV-1-infected T cell line (27) and differentiated HCMV-infected THP-1, a monocytic cell line (28). However, a comprehensive and comparative analysis of cell surface receptor regulations of HIV-1 and HCMV in primary human immune cells on a single-cell level is still lacking. Such an immune evasion “fingerprint” will facilitate the identification of novel target structures for the development of antiviral strategies and shed light on the diverse repertoire of immune evasion mechanisms exerted by the recent zoonotic (HIV-1) and the highly human-adapted (HCMV) viral pathogen.

## RESULTS

### HCMV morphologically reshapes the PM of infected macrophages.

**FIG 1:**
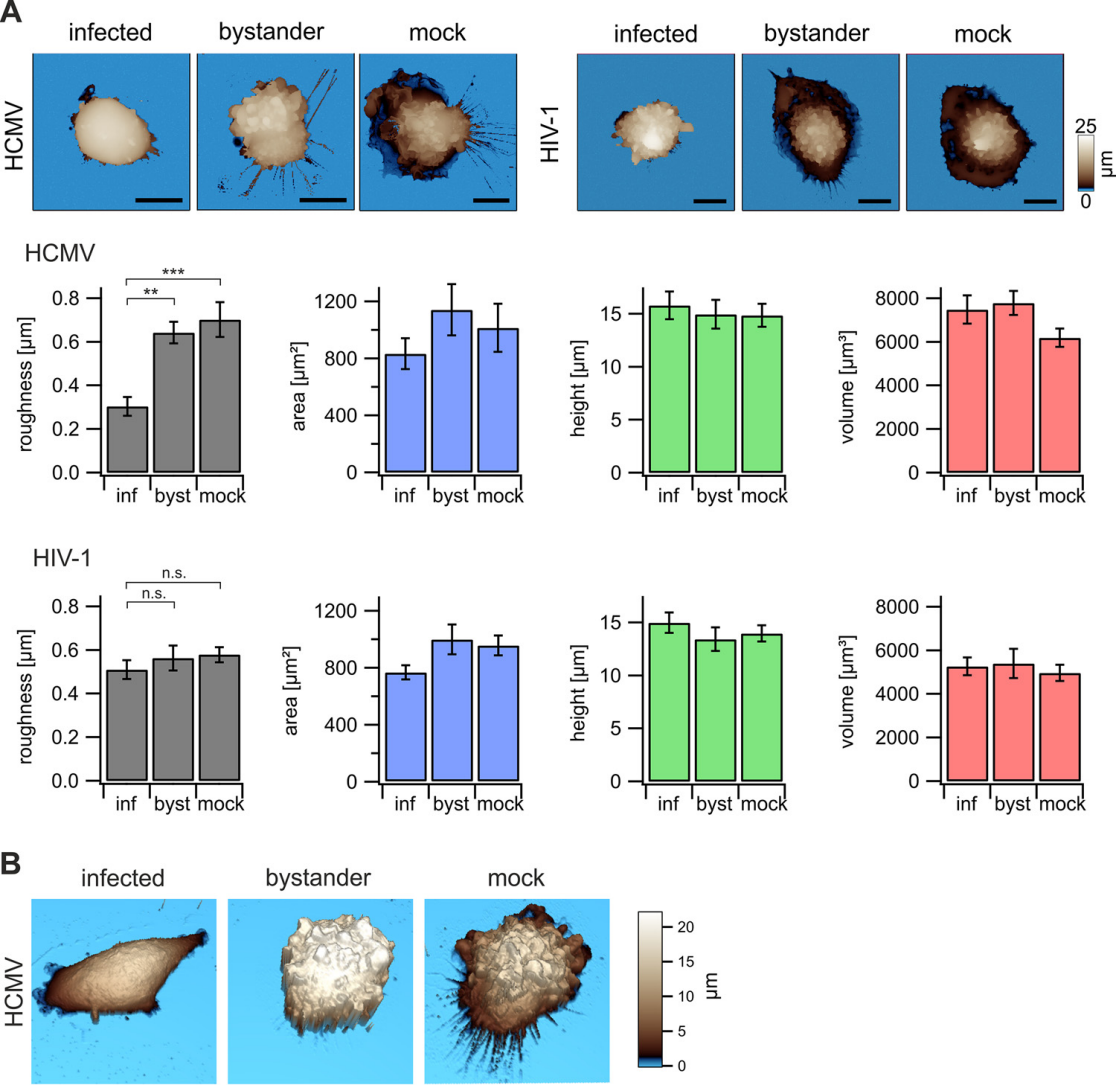
HCMV- and HIV-1-induced cytopathic effects on primary macrophages. Macrophages were either HCMV infected (TB40E-delUL16-eGFP) for 90 min or pretreated with Vpx^+^ VLPs and subsequently infected with HIV-1 (NL4.3) for 6 h (see Materials and Methods for details). At 4 dpi, the cells were fixed with 2% PFA and (A) topography images were taken with the SICM technique. *n* = 3 for each infection with 5 cells per cell population. Representative images are shown. Bars, 15 μm. Surface parameters like roughness, area, height, and volume were calculated as described in Materials and Methods. Values are means ± standard errors of the means (SEM). Significance was tested using a Tukey test. ***, *P* < 0.001; **, *P* < 0.01; n.s., not significant. (B) 3D SICM reconstructions of HCMV-infected macrophage cultures from panel A.

Our first aim was to assess on a macromolecular scale if HCMV or HIV-1 reshapes or reorganizes primary human macrophages in general or the plasma membrane in particular. Scanning ion conductance microscopy (SICM) was chosen as label- and contact-free imaging technology that preserves the native structure of cells and is applied to visualize the topography of fixed and living cells (29, 30). Taking advantage of viral strains that express green fluorescent protein (GFP) upon infection allowed us to specifically discriminate infected (GFP^+^) macrophages from bystander (GFP^−^) macrophages, i.e., macrophages in the same cell culture dish that were challenged with the virus but did not become productively infected. We found that area, height, and volume of HCMV-infected macrophages remained unaltered (Fig. 1A). In contrast, the surfaces of primary macrophages infected with HCMV were smoother than those of mock-infected and bystander macrophages (Fig. 1A and B). Conversely, HIV-1 infection did not result in any detectable alterations of roughness or any other topographic factor assessed (Fig. 1A).

### Analysis of cell surface receptor modulation in HCMV- and HIV-1-infected macrophages.

**FIG 2:**
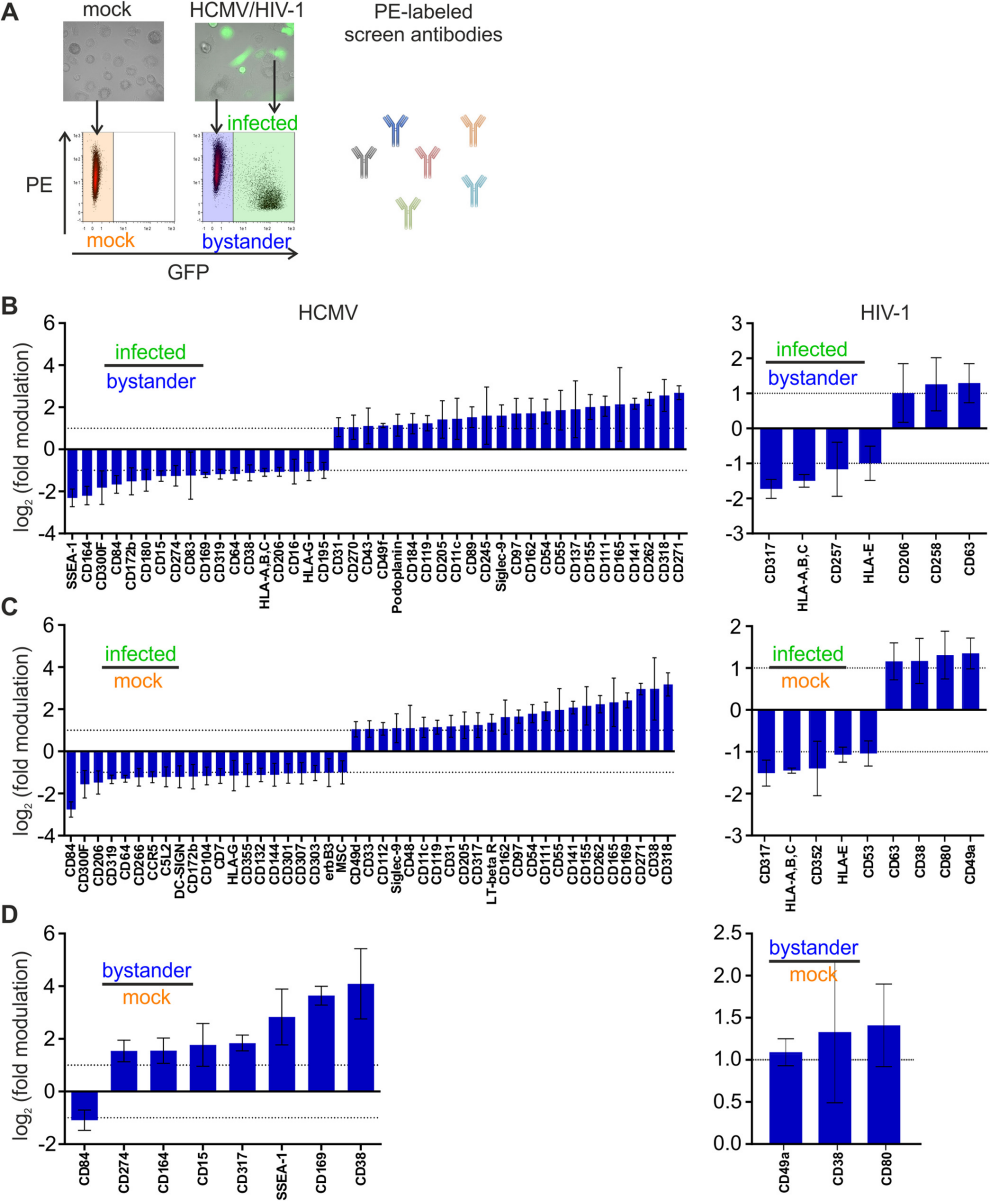
Cell surface receptors modulated in human macrophages upon HCMV or HIV-1 infection. Primary human macrophages were either mock infected or infected with TB40E-delUL16-eGFP for 90 min at 37°C. For HIV-1 infection, macrophages were pretreated with Vpx^+^ VLPs for 2 h. Subsequently, they were either mock infected or infected with the R5-tropic pBR-NL4.3 V3 92th014.12-IRES-eGFP (VSV-G pseudotyped) for 6 h. At 2 dpi, the cells were harvested, stained with 332 PE-labeled antibodies against surface receptors, and analyzed by FACS. Infected cells were discriminated by GFP expression. (A) Schematic workflow of the FACS-based screening. PE MFI of different populations (mock infected, bystander, and infected) was analyzed using FACS. (B to D) Means of log_2_[ratios (infected/bystander)] ± standard deviations (SD) of log_2_[ratios (infected/bystander)] (B), log_2_[ratios (infected/mock)] ± SD of log_2_[ratios (infected/mock)] (C), and log_2_[ratios (bystander/mock)] ± SD of log_2_[ratios (bystander/mock)] (D). *n* = 4 (HCMV) or 3 (HIV-1). Hits that fulfill all criteria (detectable expression, ≥2-fold modulation, and *P* < 0.05) are depicted in the graphs. The dashed lines at ±1 represent the criterion of ±2-fold modulation. Statistical analysis was done as indicated in Materials and Methods.

We next analyzed changes at the PM induced by HCMV and HIV-1 on a molecular level. For this, we applied a medium-throughput flow cytometry-based screen to get an unbiased and comprehensive overview of macrophage cell surface receptors being regulated by the two viruses. Mock-infected and virus-infected macrophage cultures were stained with 332 phycoerythrin (PE)-labeled cell surface receptor antibodies in a 96-well plate format and subsequently analyzed by fluorescence-activated cell sorting (FACS) (HCMV raw data are in Table S1; HIV-1 raw data are in Table S2). The usage of GFP reporter viruses allowed discrimination of productively infected (GFP^+^) from bystander (GFP^−^) macrophages in one measurement (Fig. 2A). Hits were defined by the following criteria: (i) in order to include only receptors expressed on macrophages, mean fluorescence intensity (MFI) had to be higher than 2 (∼3-fold that of the respective isotype control); (ii) receptors had to be regulated ≥2-fold; and (iii) *P* had to be <0.05.

10.1128/mBio.01770-21.5 TABLE S1 Primary FACS-data of HCMV receptor screen. MFI values, normalization, and analysis (log_2_-fold modulation) of four independent receptor screens conducted in primary HCMV-infected macrophages. Download Table S1, XLSX file, 0.2 MB. Copyright © 2021 Businger et al. 2021 Businger et al. https://creativecommons.org/licenses/by/4.0/ This content is distributed under the terms of the Creative Commons Attribution 4.0 International license.

10.1128/mBio.01770-21.6 TABLE S2 Primary FACS data of HIV receptor screen. MFI values, normalization, and analysis (log_2_-fold modulation) of three independent receptor screens conducted in primary HIV-infected macrophages. Download Table S2, XLSX file, 0.2 MB. Copyright © 2021 Businger et al. 2021 Businger et al. https://creativecommons.org/licenses/by/4.0/ This content is distributed under the terms of the Creative Commons Attribution 4.0 International license.

Applying these criteria, we identified 42 receptors which are differentially expressed between bystander and HCMV-infected macrophages, 18 of which were downregulated and 24 upregulated (Fig. 2B and Table S3). Similarly, when infected macrophages were compared to mock-infected cultures, 45 receptors showed differential cell surface levels; 21 were decreased and 24 were increased (Fig. 2C). In contrast, and as expected, only few receptors (i.e., 8) differed between bystander and mock-infected cells (Fig. 2D). Of note, the cell surface expression pattern of HIV-1-infected macrophages differed in only a few receptors relative to bystander macrophages (7 receptors) or mock-infected cells (9 receptors) (Fig. 2B to D and Table S4). This basically phenocopies the results from the SICM topographical logical profiling (Fig. 1) and confirms on a molecular level that HCMV strongly dysregulates the PM of infected macrophages, whereas HIV-1-infected macrophages have a PM morphology and molecular composition resembling those of noninfected cells.

10.1128/mBio.01770-21.7 TABLE S3 Selection of receptor candidates modulated in-HCMV infected macrophages. Criteria for hit selection and selected candidate receptors: modulation, bystander versus infected, bystander versus mock infected, and infected versus mock infected. Download Table S3, XLSX file, 0.05 MB. Copyright © 2021 Businger et al. 2021 Businger et al. https://creativecommons.org/licenses/by/4.0/ This content is distributed under the terms of the Creative Commons Attribution 4.0 International license.

10.1128/mBio.01770-21.8 TABLE S4 Selection of receptor candidates modulated in HIV-infected macrophages. Criteria for hit selection and selected candidate receptors; modulation, bystander versus infected, bystander versus mock infected, and infected versus mock infected. Download Table S4, XLSX file, 0.02 MB. Copyright © 2021 Businger et al. 2021 Businger et al. https://creativecommons.org/licenses/by/4.0/ This content is distributed under the terms of the Creative Commons Attribution 4.0 International license.

HCMV-regulated receptors include HLA-A, -B, and -C and CD206, which are already known to be modulated by the virus (20, 31), thereby validating our screening procedure. CD164, CD84, and CD180 represent examples of novel HCMV-regulated immune receptors we discovered (Fig. 2). For HIV-1, the few receptors identified comprise CD317 (tetherin), an HIV-1 restriction factor that inhibits viral release, MHC-I molecules, tetraspanins (CD53 and CD63), and some other molecules. X-fold modulation was most pronounced for CD317 and MHC-I, two receptors with firmly established functions in HIV-1 biology, i.e., enhancement of virus release and suppression of the cytotoxic T lymphocyte (CTL) response against infected cells (26, 32–34).

**FIG 3:**
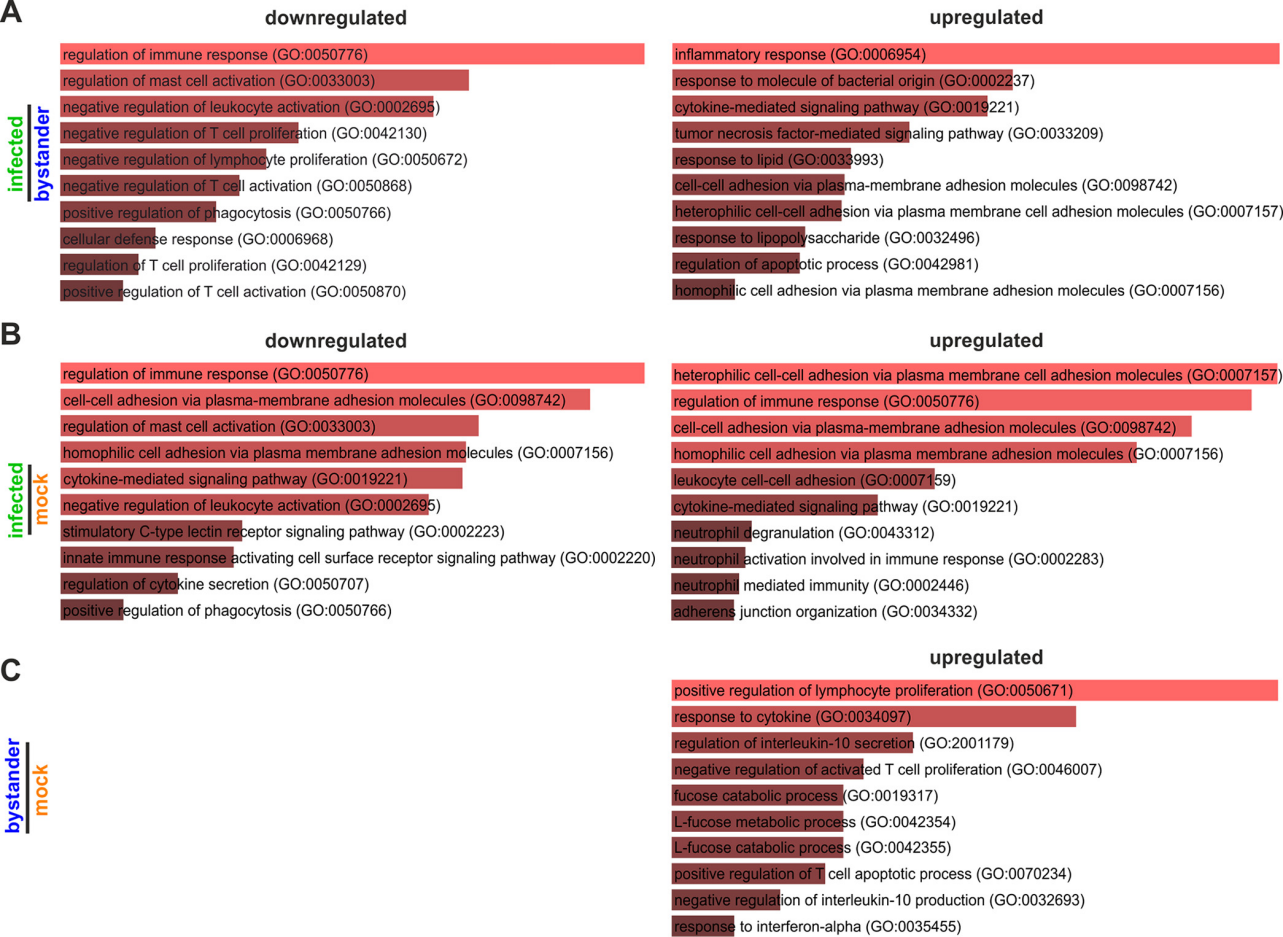
Gene ontology analysis of HCMV-mediated changes in cell surface receptor expression. Cell surface receptors which were significantly modulated by HCMV were analyzed according to their gene ontology using Enrichr (https://amp.pharm.mssm.edu/Enrichr/) (75). Shown are infected versus bystander (A), infected versus mock infected (B), and bystander versus mock infected (C), ranked by *P* value. The lowest *P* value is at the top. The graphs represent significance by bar color and length: the longer and lighter the bar, the more significant the term.

Gene ontology (GO) analysis revealed that HCMV mainly downregulates cell surface proteins that are involved in regulation of immune responses, mast cell activation and leukocyte activation (Fig. 3A and B, left). Upregulated receptors mediate inflammatory responses and cytokine-mediated signaling (Fig. 3A and B, right). Furthermore, bystander versus mock-infected cells upregulate positive mediators of lymphocyte proliferation and the response to cytokines (Fig. 3C). Altogether, even though HCMV seems to robustly blunt the immune response, productively infected as well as bystander macrophages might still be sensed and participate in the formation of a proinflammatory environment and T cell activation.

### Validation of HCMV-mediated cell surface receptor modulation in different target cells.

To follow up on the most promising hits, the list of candidate receptors was deconvoluted by excluding receptors that had already been published. To this end, primary FACS plots were checked to validate the bioinformatic analysis. Some receptors, for instance, CD97 and CD184 (CXCR4), were not further validated, since they showed differential effects upon HCMV infection of macrophages from different donors (Table S1). Some receptors were not consistently expressed among donors and hence were excluded. An example of such a receptor is CD300F, which showed variable and inconsistent surface levels in different macrophage preparations (Table S1).

**FIG 4:**
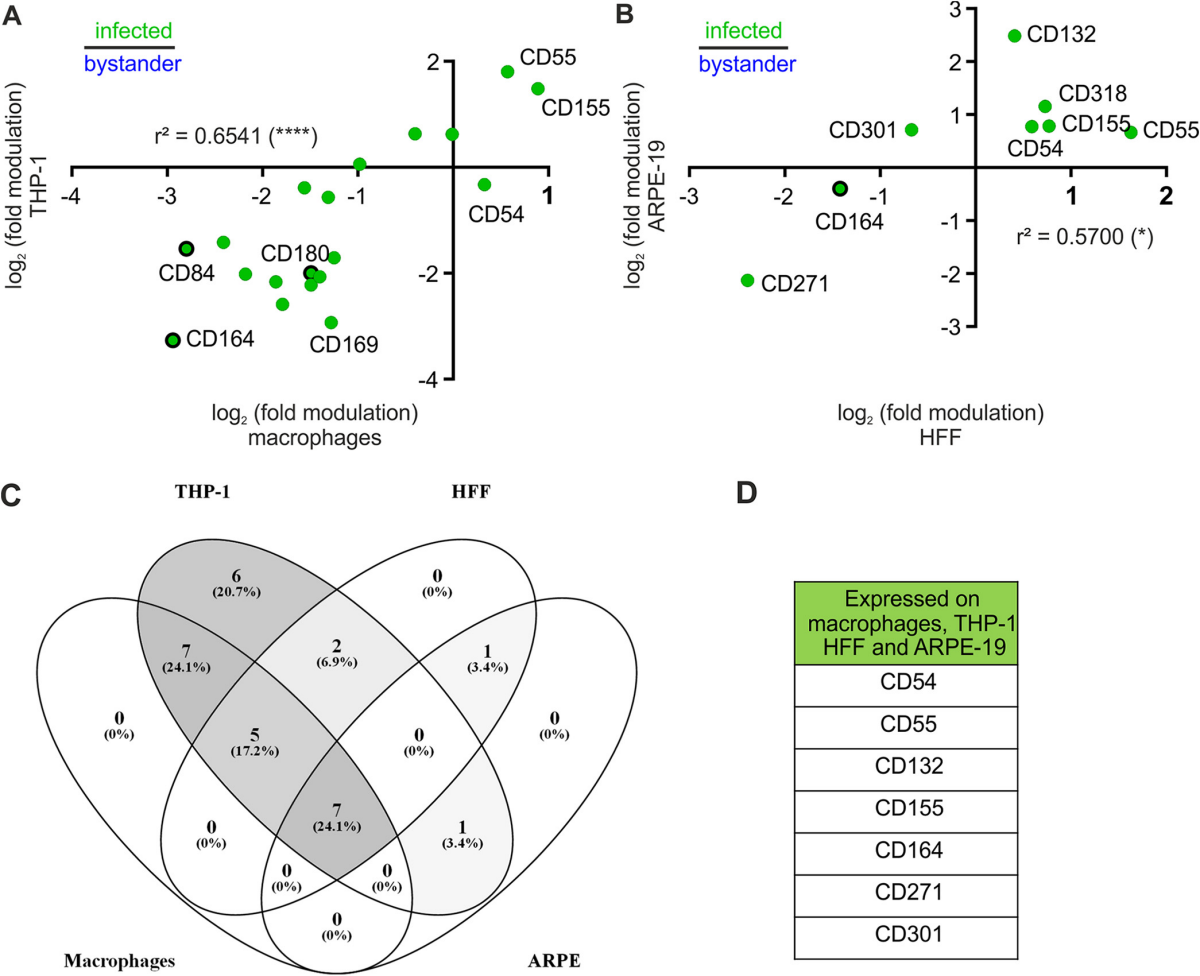
Validation of HCMV-mediated modulations on macrophages, differentiated THP-1 cells, HFF, and ARPE-19 cells. Macrophages, differentiated THP-1 cells, HFF, or ARPE-19 cells were infected with TB40-delUL16-eGFP or mock infected for 90 min. At 2 dpi, the cells were detached with Accutase and stained with 31 PE-labeled validation antibodies. (A and B) Correlation of modulation in macrophages and differentiated THP-1 cells (A) and HFF and ARPE-19 cells (B). Shown are the receptors significantly expressed in both cell types investigated. *n* = 3. *r*^2^, Pearson correlation coefficient. (C) Expressed receptors on macrophages, differentiated THP-1, HFF and ARPE-19. Numbers show how many receptors were expressed on one, two, three, or all four cell types, and corresponding percentages are displayed. (D) Receptors that are expressed and modulated on all four cell types.

**FIG 5:**
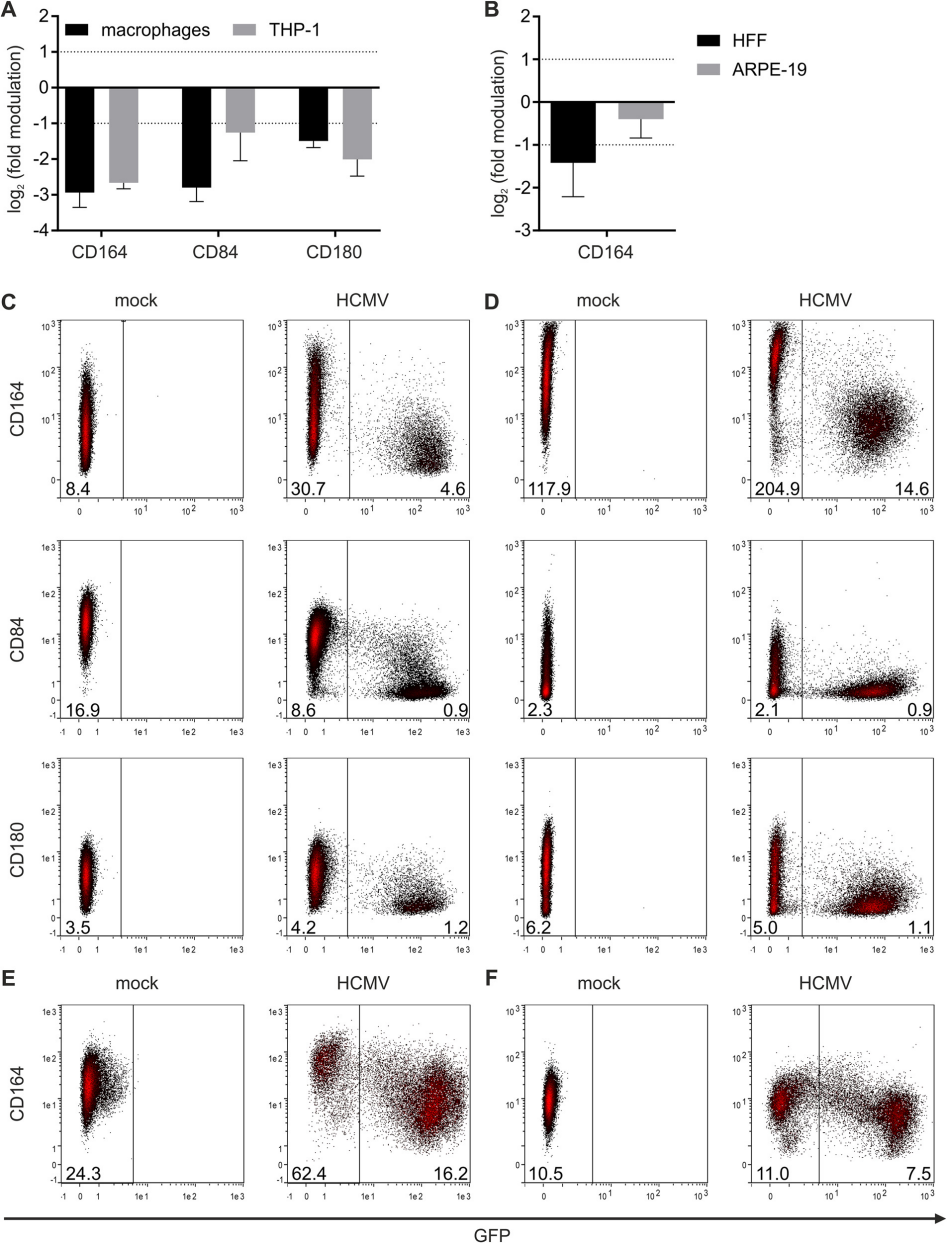
CD164, CD84, and CD180 are downregulated on HCMV target cells. (A) Macrophages and THP-1 cells and (B) HFF and ARPE-19 cells were infected with TB40E-delUL16-eGFP for 90 min. At 2 dpi, cells were harvested, stained with PE-labeled antibodies against the indicated receptors, and analyzed by FACS. Data are calculated from 3 independent experiments (data are means and SD; infected versus bystander). (C to F) Representative primary FACS plots of macrophages (C), differentiated THP-1 cells (D), HFF (E), and ARPE-19 cells (F). Numbers in plots represent the PE MFI values of the respective cell population.

After these deconvolution steps, 31 receptors modulated by HCMV were chosen for in-depth analyses (Table S5). The first step was to utilize independent antibody-clones and different HCMV-relevant target cell lines. The latter include macrophage-like cells (THP-1 cells), human foreskin fibroblasts (HFF), and epithelial cells (ARPE-19 cells). Fold modulations in macrophages correlate with fold modulations in THP-1 cells (*r*^2^ = 0.6541 and *P* < 0.0001 for infected versus bystander cells) (Fig. 4A and Fig. S1A), which demonstrates that THP-1 cells are an appropriate model cell line for primary macrophages in this setup. Again, CD164, CD84, and CD180 were identified among the receptors most downregulated on infected compared to bystander cells. In general, only a few of the 31 receptors were expressed on HFF and ARPE-19 cells (Fig. 4B, Fig. S1B, and Table S5), implying that most of the receptors are specific for immune cells in general and myeloid cells in particular. Seven receptors were expressed on all four HCMV target cells investigated (Fig. 4C and D). CD84 and CD180 were not expressed on fibroblasts and epithelial cells. CD164, however, was expressed and also downregulated from the surface of HFF and ARPE-19 cells (Fig. 4B, Fig. 5, and Fig. S2). CD55 and CD155 were upregulated on all cell lines, whereas, e.g., CD301 was differentially regulated between myeloid cells (macrophages, THP-1) and nonimmune cells (HFF and ARPE-19 cells) (Fig. S2). Altogether, we decided on three hit candidate receptors: CD164, which was downregulated from the surfaces of all cells analyzed (Fig. 5), and CD84 and CD180, which seem to be myeloid cell-specific receptors with reduced expression upon HCMV infection (Fig. 5A, C, and D and Table S4).

10.1128/mBio.01770-21.1 FIG S1 Correlation of HCMV-mediated modulations on THP-1 cells, HFF, and ARPE-19 cells. Macrophages, differentiated THP-1 cells, HFF, or ARPE-19 cells were infected with TB40-delUL16-eGFP or mock infected for 90 min. At 2 dpi, the cells were detached with Accutase and stained with 31 PE-labeled validation antibodies. (A) Correlation of modulation in macrophages and differentiated THP-1 cells and (B) HFF and ARPE-19 cells. Shown are the receptors significantly expressed in both cell types investigated. *n* = 3. *r*^2^, Pearson correlation coefficient. Download FIG S1, TIF file, 0.5 MB. Copyright © 2021 Businger et al. 2021 Businger et al. https://creativecommons.org/licenses/by/4.0/ This content is distributed under the terms of the Creative Commons Attribution 4.0 International license.

10.1128/mBio.01770-21.2 FIG S2 Regulation of receptors on HCMV target cells. (A) Macrophages, (B) differentiated THP-1, (C) HFF and (D) ARPE-19 cells were infected with TB40E-delUL16-eGFP for 90 min. At 2 dpi cells were harvested, stained with PE-labeled antibodies against the receptors indicated and analyzed by FACS. Shown are primary FACS plots representative of three experiments. Numbers in plots represent the PE MFI of the respective cell population. Download FIG S2, TIF file, 2.6 MB. Copyright © 2021 Businger et al. 2021 Businger et al. https://creativecommons.org/licenses/by/4.0/ This content is distributed under the terms of the Creative Commons Attribution 4.0 International license.

10.1128/mBio.01770-21.9 TABLE S5 Validation of HCMV-modulated candidate receptors in different cell lines. MFI values from the primary macrophage screen, ratios, and mean expression levels. MFI values, ratios, and mean expression levels from the validation screen with an independent antibody panel in primary macrophages (*n* = 4), THP-1 cells (*n* = 3), HFF (*n* = 3) and ARPE cells (*n* = 3). Download Table S5, XLSX file, 0.2 MB. Copyright © 2021 Businger et al. 2021 Businger et al. https://creativecommons.org/licenses/by/4.0/ This content is distributed under the terms of the Creative Commons Attribution 4.0 International license.

### HCMV decreases cell surface expression of CD164, CD84, and CD180 on a transcriptional level.

**FIG 6:**
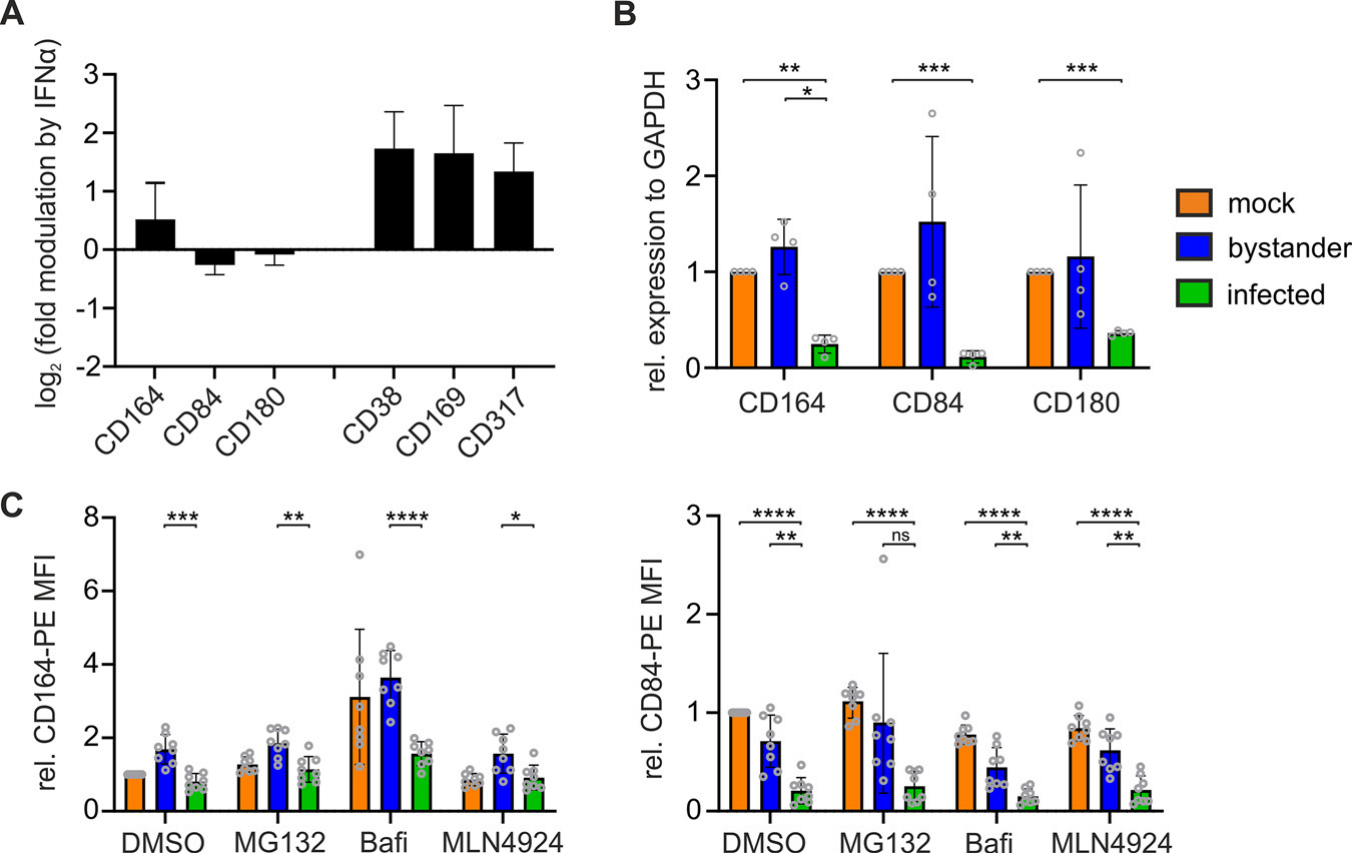
HCMV downmodulates CD164, CD84, and CD180 by transcriptional regulation. (A) Macrophages were treated with 10 ng ml^−1^ IFN-α-2a or IFN-γ for 48 h, stained with PE-labeled validation antibodies, and analyzed by FACS. (B) At 4 dpi, TB40-delUL16-eGFP-infected macrophages were sorted into mock-infected, bystander, and infected populations according to their GFP expression. Relative RNA expression of CD164, CD84, and CD180 was determined by qRT-PCR. *n* = 4. (C) At 24 h after infection with TB40E-delUL16-eGFP, the cells were treated with DMSO, 1 μM MG132, 500 nM bafilomycin A1, or 0.1 μM MLN4924 for 6 h. Subsequently, the cells were collected and stained for CD164 or CD840. *n* = 8. Data are means ± SD. Significance was tested with two-way analysis of variance (ANOVA) with Tukey’s comparison test. ****, *P* < 0.0001; ***, *P* < 0.001; **, *P* < 0.01; *, *P* < 0.05.

HCMV efficiently blunts the type I interferon (IFN) response in infected cells (35, 36). This blunting could represent a mechanism by which HCMV reduces the expression of interferon-induced cell surface receptors. To assess this, we treated primary macrophages with IFN-α or IFN-γ and analyzed which of the 31 receptors were induced by interferon in primary human macrophages (Fig. S3). As expected, expression of CD38, CD169, and CD317 as canonical interferon-stimulated genes (ISGs) increased upon IFN-α treatment (32, 37–39), whereas cell surface levels of CD164, CD84, and CD180 were not increased by IFN-α or IFN-γ (Fig. 6A). Hence, disruption of the IFN response is not the underlying cause of reduced cell surface expression of these receptors upon HCMV infection. Furthermore, productive HCMV infection is essential to reduce receptor cell surface levels, as inactivation of the virus by UV-C abrogates viral gene expression as well as receptor modulation (Fig. S4).

10.1128/mBio.01770-21.3 FIG S3 Cell surface regulation of hit receptors by IFN-α or IFN-γ treatment. Macrophages were treated with 10 ng ml^−1^ IFN-α-2a or IFN-γ for 48 h, stained with PE-labeled validation antibodies, and analyzed by FACS. *n* = 4. Data are means ± SD. Download FIG S3, TIF file, 0.3 MB. Copyright © 2021 Businger et al. 2021 Businger et al. https://creativecommons.org/licenses/by/4.0/ This content is distributed under the terms of the Creative Commons Attribution 4.0 International license.

10.1128/mBio.01770-21.4 FIG S4 UV-inactivation abrogates cell surface receptor modulation by HCMV. (A) Infection of HFF to validate the UV-inactivation procedure by loss of productive infection and induction of the ISG CD317 or (B) macrophages with nontreated (NT) or UV-C-inactivated (UV, 2.4 mW/cm^2^; 5 min) TB40E-delUL16-eGFP. At 2 dpi, cells were harvested, stained with PE-labeled antibodies against the receptors indicated, and analyzed by FACS. (A and B) Primary FACS plots of one representative experiment. Numbers in the dot plots indicate the respective PE-MFI of the gated cell population. (C) Column diagram of 6 independent biological replicates of macrophage infection (mean ± SD). Download FIG S4, TIF file, 1.9 MB. Copyright © 2021 Businger et al. 2021 Businger et al. https://creativecommons.org/licenses/by/4.0/ This content is distributed under the terms of the Creative Commons Attribution 4.0 International license.

Diminished expression of cell surface receptors could be achieved by transcriptional silencing. Therefore, we sorted HCMV-infected macrophages according to their GFP expression and assessed RNA levels of CD164, CD84, and CD180 by quantitative reverse transcription-PCR (qRT-PCR). Of note, CD164, CD84, and CD180 transcripts were significantly reduced in HCMV-infected macrophages compared to mock-infected controls or bystander cells (Fig. 6B).

Common alternative pathways for cell surface receptor regulation are internalization and degradation by the proteasome or lysosome. To test for this, we treated macrophages with inhibitors of proteasomal (MG132 and MLN4924) or lysosomal (bafilomycin A) degradation and assessed cell surface regulation of CD164 and CD84 (Fig. 6C). As expected, none of the treatments rescued cell surface expression of the receptors, corroborating the idea that HCMV induces transcriptional silencing to reduce expression of CD164, CD84, and CD180.

### Canonical HCMV immune evasion gene regions are not involved in downregulation of CD164, CD84, and CD180.

**FIG 7:**
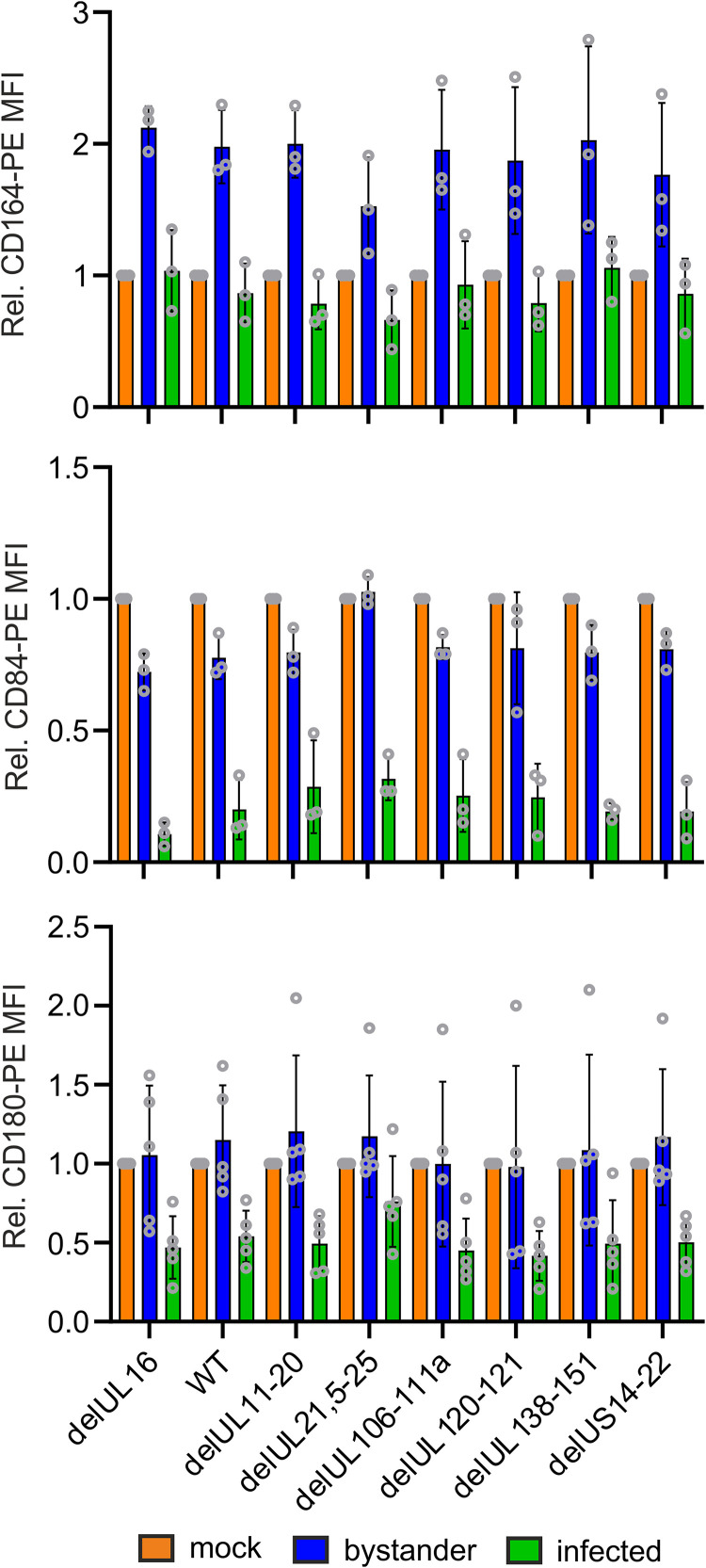
CD164, CD84, and CD180 are not downregulated by classical viral immune evasion proteins. Macrophages were infected with GFP-expressing HCMV deletion mutants for 30 min, followed by a 30-min spinoculation. At 2 dpi, the cells were harvested and stained with PE-labeled antibodies, and receptor expression was measured by flow cytometry. *n* = 3 (CD164 and CD84) or 5 (CD180). Data are means ± SD. Data were normalized to relative receptor expression of the mock-infected cells.

To get first insights into the viral proteins involved in dysregulation of CD164, CD84, or CD180 by HCMV, we infected macrophages with HCMV variants harboring a series of systematical deletions in specific gene regions. These deletions were introduced based on the fact that the respective regions are common immune evasion gene clusters of HCMV. Subsequently, macrophages were stained for CD164, CD84, and CD180, and cell surface modulation by the various HCMV mutants was quantified by flow cytometry (Fig. 7). Altogether, none of the deletions impaired the ability of HCMV to reduce cell surface expression of the receptors compared to the GFP-expressing delUL16 variant, which was used in previous experiments, or the corresponding wild-type (WT) virus (BAC4-GFP) (Fig. 7). Hence, HCMV genes other than those investigated here are involved in modulation of CD164, CD84, and CD180.

### Clinical HCMV isolates downmodulate CD164, CD84, and CD180 from the surfaces of primary macrophages.

**FIG 8:**
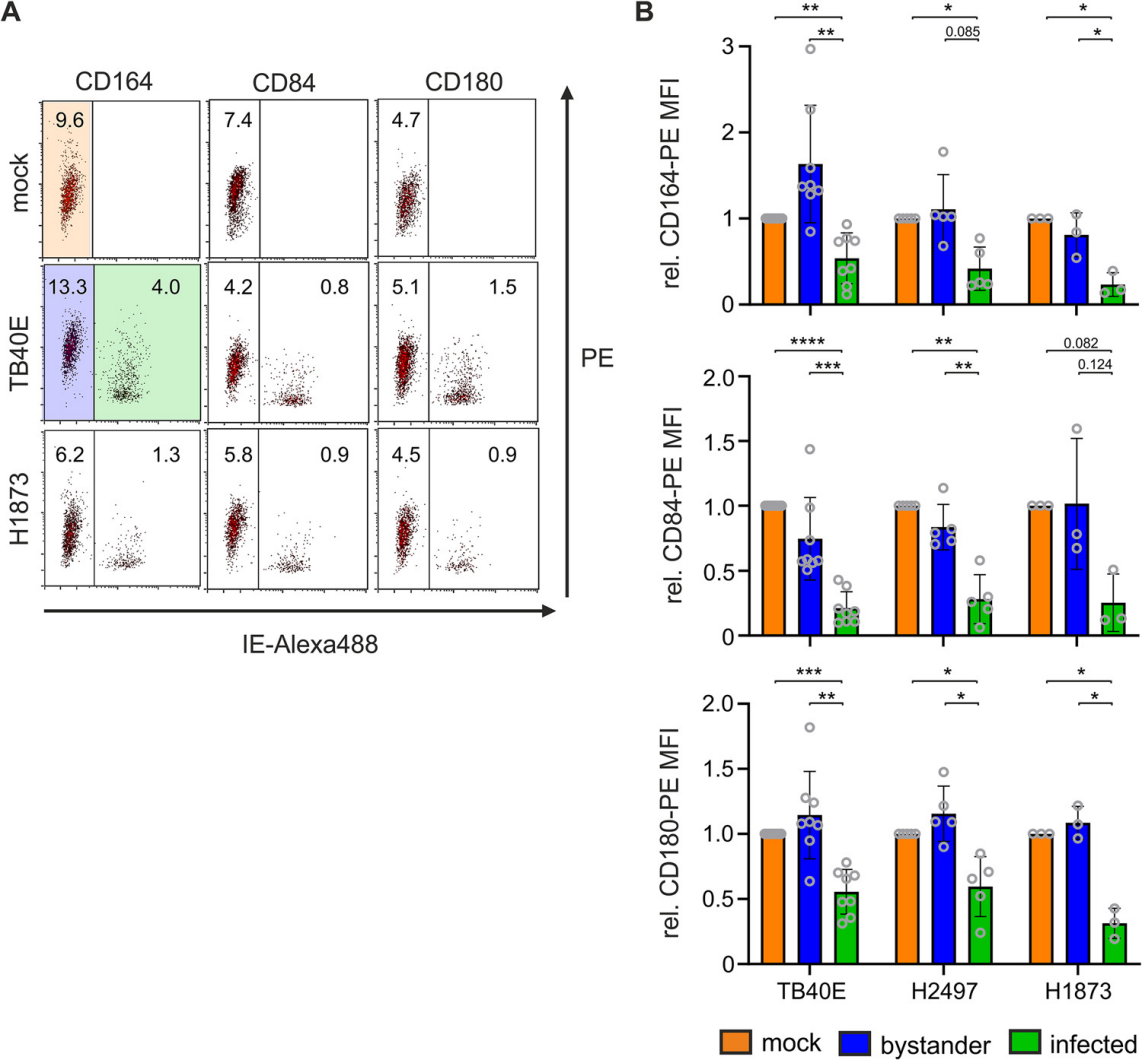
Two clinical HCMV isolates downregulate CD164, CD84, and CD180. Macrophages were infected with the cell-free supernatants of HCMV TB40E or clinical isolates H2497 and H1873 for 30 min followed by a 30-min spinoculation. At 2 dpi, the cells were harvested and stained with PE-labeled antibodies, and receptor expression was measured by flow cytometry. (A) Primary representative FACS plots from one experiment with H1873. Numbers in plots represent the PE MFI of the respective cell population. (B) Column diagram of independent biological replicates. *n* = 8 (mock, TB40E), 5 (H2497), or 3 (H1873). Data are means ± SD. Significance was tested with two-way ANOVA with mixed-effects analysis. ****, *P* < 0.0001; ***, *P* < 0.001; **, *P* < 0.01; *, *P* < 0.05. Data were normalized to relative receptor expression of the mock-infected cells.

Lab-adapted viral strains might accumulate mutations and could potentially acquire or lose functions that are relevant *in vivo* but not in cell culture. We hence assessed modulation of CD164, CD84, and CD180 by two clinical HCMV isolates. HCMV H2497 and H1873 were isolated from amniotic fluid and subsequently passaged on ARPE-19 cells to maintain viral tropism for macrophages. HCMV H2497 and H1873 downregulated CD164, CD84, and CD180 in a manner which is comparable to (H2497) or even more pronounced than (H1873) that of the lab-adapted strain TB40E WT (Fig. 8). This suggests that there is positive selection on these functions *in vivo* and indicates that downregulation of these factors is a potentially important function for HCMV replication and persistence in the infected human host.

## DISCUSSION

The human cytomegalovirus is highly adapted to its host, causes lifelong latency, and encodes a series of immunomodulatory proteins to ensure persistence. Similarly, HIV-1 causes chronic infections in humans and has evolved efficient strategies to evade the antiviral immune response. We assessed remodeling of the PM by both viruses to potentially elucidate novel mechanisms of viral immune evasion.

Contact-free scanning ion conductance microscopy (SICM) demonstrated a “flattened” surface of macrophages infected with HCMV, in contrast to those infected with HIV-1. Strikingly, this phenotype was consistent on a molecular level, as HCMV induced profound changes in cell surface receptor expression levels, whereas HIV-1 modulated only a very few receptors on the surfaces of macrophages. These differential phenotypes might highlight opposing strategies of viral immune evasion in this cell type. Whereas HIV-1 hides in macrophages that are morphologically and phenotypically similar to noninfected cells, HCMV reorganizes the PM to avoid immune-cell signaling and clearance. Hence, HIV-1 might have evolved to modulate only the most important antiviral pathways, i.e., CD317 (tetherin) and HLA-A, -B, and -C (MHC-I). Of note, few studies have assessed HIV-1-induced cell surface receptor regulation in macrophages, whereas a bevy of studies as well as a proteomic screen reported massive remodeling of the PM in an HIV-1-infected CD4^+^ T cell line (7, 21, 24, 27). In this cell type, HIV-1 assembles at the PM and is highly cytopathic.

In contrast, the PM was extensively remodeled by HCMV. HCMV replicates productively in macrophages, as it has evolved efficient countermeasures against SAMHD1 (40–43). Identification of known receptors, such as HLA-A, -B, and -C or CD206, which are downregulated by productive HCMV infection as well as the upregulation of, e.g., CD55 demonstrated the validity of our screening procedure (31, 44–48). Furthermore, TB40E delUL16-eGFP, in addition to the deletion in UL16, harbors a frameshift in UL141, which leads to nonfunctional pUL141, pUL144, and pUL145 (TB40E-Bart) (23, 49). This virus strain enhanced the cell surface expression of CD155, which is in accordance with our findings (Fig. S2).

We verified receptor modulations in different HCMV target cells using independent antibody clones. In this analysis, from the 42 hits initially screened to be down- or upregulated by HCMV in macrophages versus bystander cells, we identified CD84 and CD180 specifically downregulated in myeloid cells and CD164 which was downregulated from the surface of all cell types tested. Importantly, these receptors are not induced by IFN; hence, transcriptional silencing of their expression is not associated with the well-known ability of HCMV to blunt the IFN response (36).

CD180 is exclusively expressed on immune cells, which we confirmed by our findings. CD180 (RP105) belongs to the Toll-like receptor (TLR) family and is considered to be a TLR4 homologue. CD180 mediates macrophage and B-cell activation and enhances the release of inflammatory cytokines (50–53). Hence, reduction of CD180 by HCMV could represent a strategy to avoid these antiviral responses.

CD84, which is SLAM family member 5, is known to be involved in the “regulation and interconnection of the innate and adaptive immune response” (reviewed in reference 54). Moreover, CD84 contributes to monocyte activation and cytokine secretion of human monocyte-derived dendritic cells (55, 56). In the context of murine CMV (MCMV) infection, CD84 is also downregulated on infected macrophages (57). Thus, HCMV and MCMV use conserved immune evasion strategies, and the mouse model could be exploited to reveal the importance of this receptor regulation for CMV infection and spread *in vivo*.

CD164 was more efficiently downregulated from the surfaces of infected macrophages than bystander cells. CD164 (sialomucin core protein 24 or endolyn) is a cell adhesion molecule involved in proliferation and migration of hematopoietic progenitor cells (58, 59). Intriguingly, a mutated form of CD164 seems to be associated with hearing loss. Nyegaard and colleagues demonstrated that a mutated form of CD164 is sequestered from the cell surface into the cytosol, where it mislocalizes to endoplasmic vesicles (60). Since congenital HCMV infection is the leading infectious cause of hearing loss (61), it is tempting to speculate that CD164 might be a central player in the processes underlying HCMV pathogenesis. This is further emphasized by the fact that CD164 is expressed in all cell types tested and downregulated upon HCMV infection. Furthermore, CD164 is an HIV-1 restriction factor (62), and HCMV might transcriptionally silence its expression to antagonize potential antiviral activity exerted against HCMV.

While the flow cytometry single-cell approach of our study and the use of various HCMV target cell lines to verify our results is an asset, there are also limitations which need to be considered. First of all, our study is not holistic, as it is limited and biased by the antibody panel used. Second, PM alterations were measured at a single time point (48 h postinfection [hpi]). Because of this, we might have missed differential and dynamic receptor modulations that occur in a time-dependent manner.

Nevertheless, taken together, our profiling of the PM of HIV-1 and HCMV-infected macrophages revealed differential strategies of human-pathogenic viruses to evade the antiviral immune response. Furthermore, we identified novel cell surface receptors that are targeted by HCMV, most likely by transcriptional shutoff. These receptors might represent novel previously unprecedented strategies of HCMV immune evasion and help to shed light on the complex coevolution of the human immune system and HCMV.

## MATERIALS AND METHODS

### Cell culture.

Primary human macrophages, THP-1 cells (ATCC TIB-202), HFF (ATCC SRC-1041), ARPE-19 cells (ATCC CRL-2302), and HEK 293 T cells (DSMZ ACC635) were cultured at 37°C with 5% CO_2_. Primary human macrophages were prepared and differentiated as described below and maintained in macrophage medium (RPMI supplemented with 4% human AB serum, 2 mM l-glutamine, 100 μg ml^−1^ penicillin-streptomycin, 1 mM sodium pyruvate, 1× nonessential amino acids, and 0.4× minimal essential medium [MEM] vitamins). HFF, ARPE-19 cells, and 293 T cells were passaged and cultured in DMEM containing 5 or 10% fetal calf serum (FCS), respectively, with 2 mM l-glutamine and 100 μg ml^−1^ penicillin-streptomycin. THP-1 cells were maintained in RPMI supplemented with 10% FCS, 2 mM l-glutamine, and 100 μg ml^−1^ penicillin-streptomycin. THP-1 cells (10^6^) were differentiated with 30 ng ml^−1^ phorbol myristate acetate for 24 h at 37°C in a six-well plate.

### Isolation and differentiation of primary human macrophages.

Macrophages were generated from buffy coats of healthy blood donors who gave informed consent for the use of blood-derived products for research purposes. We do not collect data concerning age, gender, or ethnicity, and we comply with all relevant ethical regulations approved by the ethics committee of the University Hospital Tübingen (IRB no. 507/2017B01). All buffy coat donations are received in pseudonymous form and chosen randomly. Peripheral blood mononuclear cells were isolated from buffy coats by Biocoll density gradient centrifugation and differentiated for 3 days by plastic adherence in macrophage medium. After 3 days, nonadherent cells were removed by washing and the macrophages were further differentiated for 4 days with macrophage medium.

### Viruses and clinical isolates.

HCMV TB40 WT is a laboratory HCMV strain derived from the clinical isolate TB40 and adapted to endothelial cells (63). TB40-delUL16-eGFP is a derivative of the TB40 strain in which the majority of the open reading frame UL16 has been replaced by the open reading frame of enhanced GFP (eGFP), essentially as described in detail for a homologous mutant of strain AD169 (64). TB40-delUL16-eGFP expresses eGFP under the control of the early UL16 promoter and has previously been used for FACS analyses (65). HCMV deletion mutants lacking specific open reading frames (Fig. 7) were generated by applying bacterial artificial chromosome (BAC)-based mutagenesis (66) to the HCMV BAC TB40/E-EGFP (67), which carries the genome of the TB40/E virus as well as the EGFP reporter protein. To delete selected open reading frames, they were replaced by a kanamycin resistance (Kan^r^) cassette. In brief, the Kan^r^ gene was PCR amplified using the template plasmid pOri6K-F5 and primers that provide sequences (45 to 50 nucleotides [nt]) homologous to the insertion site within the BAC. Escherichia coli strain DH10B carrying the TB40/E-EGFP BAC as well as plasmid pKD46 (68) was induced with l-arabinose in order to express the recombination proteins red-α, -β, and -γ and to become recombination proficient. Subsequently, the bacteria were electroporated with the Kan^r^ PCR product, and resistant bacterial clones were selected on agar plates containing kanamycin and chloramphenicol. Correct modification of the HCMV BACs was verified by restriction analysis of BAC DNA. To reconstitute the deletion mutants, HFF were transfected with BAC DNA as described elsewhere (66).

The clinical HCMV isolates H2497 and H1873 were isolated from amniotic fluid of two HCMV-infected but antiviral-naive pregnant women and primarily isolated from ARPE-19 cells without any prior fibroblast adaption (69).

### Generation of HCMV stocks.

To generate HCMV stocks, HFF cells were infected with either TB40-delUL16-eGFP, BAC4-GFP, or the corresponding deletion mutants. Infectious supernatant was collected at 5 to 7 days postinfection (dpi) and subsequently cleared from cells and cellular debris by centrifuging for 10 min at 3,200 × *g*.

### HCMV infection assays.

For infection experiments, macrophages, differentiated THP-1 cells, HFF, or ARPE-19 cells were seeded on a six-well plate the day before infection. Macrophages or differentiated THP-1 cells were preincubated with DMEM containing 5% FCS for 1 h at 37°C. Cells were infected with TB40-delUL16-eGFP or mock infected for 90 min at 37°C. Then, the medium was changed to fresh cell-specific medium and the cells were further incubated at 37°C. For infection with the deletion mutants and the clinical isolates, macrophages were pretreated with DMEM containing 5% FCS for 2 h at 37°C and then infected for 30 min at 37°C. Thereafter, the virus was diluted and spinoculated for 30 min at 1,000 × *g* at 34°C. The medium was changed 90 min later, and the cells were further incubated at 37°C. At the indicated time points, cells were collected by Accutase treatment and further processed for FACS analysis. Since HCMV stocks were used fresh, back-titration on HFFs was done to evaluate the multiplicity of infection (MOI). Primary macrophages and THP-1 cells were infected with an MOI between 5 and 10. Examples of primary infection rates in three different macrophage donors that were infected with equal MOIs of the various HCMV stocks are presented in Table S6.

10.1128/mBio.01770-21.10 TABLE S6 Primary infection rates of HCMV-GFP variants in different macrophage donors. Macrophages were infected with HCMV-GFP mutant viruses at an MOI of 5 (Fig. 7). The absolute infection rate was measured by assessing the proportion of GFP^+^ cells by flow cytometry. Number are representative numbers from three different macrophage donors to illustrate the variability in primary infection rates, even though the same MOI were used. Correlation analysis and calculation of *P* values and Pearson coefficient (*r*) was done using GraphPad Prism. Download Table S6, XLSX file, 0.03 MB. Copyright © 2021 Businger et al. 2021 Businger et al. https://creativecommons.org/licenses/by/4.0/ This content is distributed under the terms of the Creative Commons Attribution 4.0 International license.

### HIV-1 constructs and generation of HIV-1 and VLP stocks.

To produce vesicular stomatitis virus G protein (VSV-G)-pseudotyped HIV-1 or virus-like particle (VLP) stocks, 0.45 × 10^6^ 293 T cells were seeded per well of a six-well plate 1 day in advance. 293 T cells were transfected using the calcium phosphate method with 5 μg of pBR-NL43_V3_92th014.12-IRES-GFP (12) or pSIV_Vpx+ (70) and cotransfected with pHIT60 (VSV-G) or mock transfected (VSV-G only). Six to 16 h after transfection, medium was changed and supernatants containing the HIV-1 or VLP stock were collected and cleared 24 h later.

### SICM.

SICM is a contact-free scanning probe microscopy technique which is specifically suited to determine the morphology of cells. Macrophages were seeded on a Greiner glass bottom dish with a density between 4 × 10^4^ to 8 × 10^4^ per 2 ml. For HCMV infection, macrophages were preincubated with DMEM (supplemented with 5% FCS, 2 mM l-glutamine, and 100 μg/ml penicillin-streptomycin). Macrophages were then infected with TB40E-delUL16-eGFP or left untreated (mock infected) for 90 min. To enhance HIV-1 infection of macrophages, the cells were pretreated with Vpx^+^ VLPs for 2 h. Subsequently, macrophages were infected with VSV-G-pseudotyped pBR-NL4.3 V3 92th014.12_IRES-eGFP or VSV-G only (mock infected) for 6 h. At 4 dpi, macrophages were fixed with 2% paraformaldehyde (PFA) for 20 min at room temperature, and topography images were recorded using SICM in the hopping mode. Calculations of cellular morphology parameters were done as follows: area = Σ(pixel area of the cell); height = maximum vertical position of the cell when the top 2.5% is neglected; volume = Σ(pixel area × pixel height).

Roughness of a sample’s surface is defined by the deviation from the baseline. This baseline was identified by applying a two-dimensional (2D) median filter of 19 by 19 pixels (corresponding to an area of 4.75 μm by 4.75 μm). Roughness was calculated from data points higher than 20% of the cell height to exclude artifacts. The standard deviations of the differences between data points and baseline are considered the roughness of the cell.

### HCMV and HIV-1 infection for screening procedure.

Petri dishes were used to allow macrophages to differentiate. First, cells were preincubated with DMEM containing 5% FCS, 2 mM l-glutamine and 100 μg/ml penicillin-streptomycin for 1 h. Afterwards, macrophages were either mock infected or with TB40E-delUL16-eGFP for 90 min. For HIV-1 infection, macrophages were pretreated with pseudotyped Vpx-containing VLPs for 2 h and subsequently infected with VSV-G-pseudotyped pBR-NL4.3 V3 92th014.12_IRES-eGFP or VSV-G only (mock infected) for 6 h. At 2 dpi, the cells were detached by Accutase treatment and stained with the LEGENDScreen kit (BioLegend) according to the manufacturer’s instructions.

### Statistical analysis of LEGENDScreen.

Bioinformatic analysis of the flow cytometry screen data was done by the QBiC (Quantitative Biology Center, University of Tübingen). All PE mean fluorescence intensity (MFI) values generated by the flow cytometer were converted to log_2_(MFI). To achieve a comparable distribution of these values in all screens, values were normalized using the quantile normalization method that is regularly used in the context of analyzing array data (71). The mean of modulation (*M*) was assessed as follows:

$$M={\text{log}}_{2}\frac{1}{n}\frac{\sum_{i=1}^{n}x_{i}}{\sum_{i=1}^{n}y_{i}}$$

where *x* is not equal to *y* and *n* is the number of PE-labeled antibodies in the screen. Receptor modulation analysis was performed with linear mixed models (nlme R package [https://cran.r-project.org/web/packages/nlme/index.html]). The effect of the condition (mock infected, bystander, and infected) on receptor expression was modeled as follows: MFI ≈ condition + random(donor/screen type), where the condition has the main effect and the screen type nested within one donor is a random factor. Infected and bystander cells were measured from the same culture dish, whereas mock-infected cells represent an independent culture. Therefore, the model accounts for this matching such that infected and bystander cells have screen type “paired” while mock-infected cells are “single” for each donor. *Post hoc* analysis was performed with Tukey's honestly significant difference method to get the actual pairwise differences among mock-infected, bystander, and infected cells. This standard procedure accounts for multiple comparisons among the three conditions and reports adjusted *P* values according to (72). Correction for multiple-hypothesis testing (multiple receptors) was done by the *q*-value method at a false discovery rate (FDR) of <0.05 (*q* value is a pFDR analogue of the *P* value [73, 74]). All calculations are summarized in Tables S1 to S5.

### FACS staining.

Cells were collected with Accutase and directly surface stained using anti-CD164, anti-CD84, and anti-CD180 (Miltenyi) for 20 min at 4°C. After two washing steps with FACS buffer (500 × *g* for 6 min), cells were fixed with 2% PFA for 10 min at room temperature. Cells were again washed and finally resuspended in FACS buffer. Cells infected with GFP-containing viruses were directly analyzed by FACS. Macrophages infected with the clinical isolates or TB40E WT were further stained for the immediate early (IE) HCMV protein by intracellular staining. For this, the macrophages were permeabilized with 80% acetone in water for 7 min at room temperature, washed again twice, and blocked with 10% FCS in PBS for 30 min at RT. Subsequently, cells were stained with IE1-Alexa488 conjugated anticytomegalovirus antibody (clone 8B1.2; Alexa Fluor 488 [analyte specific reagent] from Merck; dilution 1:200) antibody for 1 h at room temperature. After a final washing step, the cells were analyzed using a MACSQuant VYB (Miltenyi). FACS analysis was performed with MACS Quantify software (Miltenyi) and Flowlogic (Miltenyi–Inivai). As a control for ISG induction, we stained CD38-PE (Miltenyi), CD169-PE (Miltenyi), and CD317-PE (Miltenyi) on the surface before fixing the cells for 30 min at 4°C. For verification of screening hits, we ordered, upon availability, a set of independent antibodies and clones from Miltenyi. Table 1 presents information about the antibodies used.

**TABLE 1:**
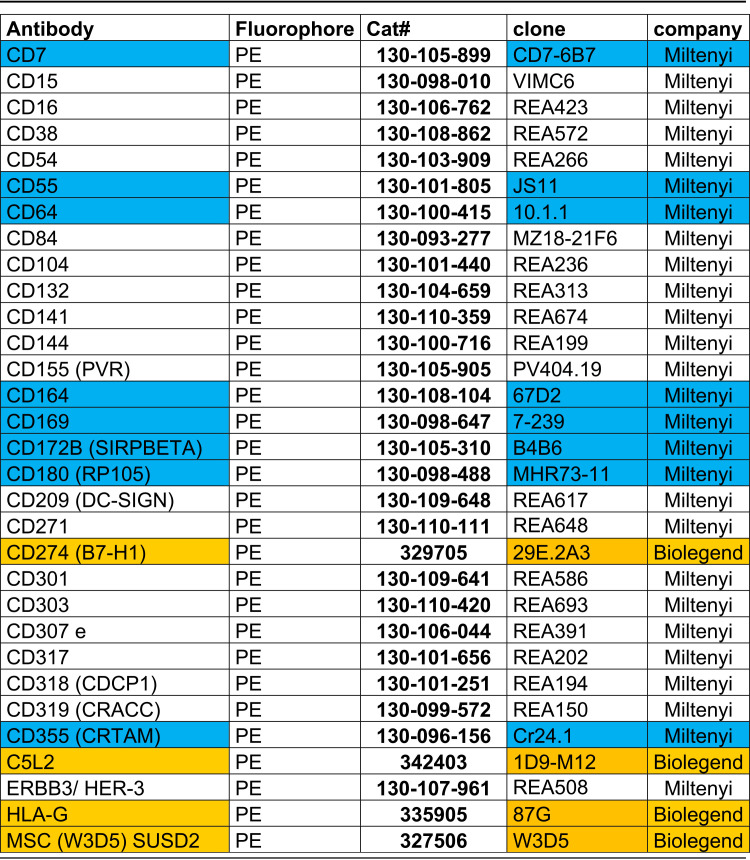
Antibodies used for FACS staining^*a*^

a Blue, same clone as in the LEGENDScreen but antibody from Miltenyi; orange, reordered same clone and same company as in the LEGENDScreen; all others are different clones.

### IFN treatment.

Macrophages were differentiated in macrophage medium (see above; with 4% human AB serum). After differentiation, the cells were treated with 10 ng ml^−1^ IFN-α-2a (PBL Assay Science; catalog no. 11100-1) or IFN-γ (ImmunoTools; catalog no. 11343534) for 48 h, stained with the 31 validation antibodies, and fixed with 2% PFA.

### Inhibitor treatment experiments.

Macrophages were seeded at 0.2 × 10^6^ per well in a six-well plate. The next day, cells were infected with TB40-delUL16-eGFP for 2 h. Twenty-four hours later, cells were inoculated with dimethyl sulfoxide (DMSO), 1 μM MG132 (AdipoGen Life Sciences), 500 nM bafilomycin A1 (AdipoGen), or 0.1 μM MLN4924 (Chemgood) for 6 h. Afterwards, macrophages were collected and stained for FACS analysis.

### qRT-PCR.

To analyze specific transcripts using qRT-PCR, HCMV-infected macrophages (TB40-delUL16-eGFP-infected macrophages) were sorted into mock-infected, bystander, and infected populations according to their GFP expression. Cells were lysed in RLT buffer (Qiagen) with β-mercaptoethanol and RNA was extracted with the RNeasy minikit (Qiagen) following the manufacturer’s instructions. cDNA was generated using the QuantiTect reverse transcription kit (Qiagen). Subsequently, qRT–PCR was done with the LightCycler 480 SYBR green I master mix using specific primers for CD164 (forward, GTGAAGGTCGAAACAGCTGC; reverse, CTGTCGTGTTCCCCACTTGA), CD84 (forward, AATGGCATCTGTGAACAGCA; reverse, ATTCTGGACTCTGCTGGCTG), CD180 (forward, GCTTCTTTTGGGTGGTGCTG; reverse, TCATGAGTCTGCTGAAGGTTCT), and GAPDH (forward, TGCACCACCAACTGCTTAGC; reverse, GGCATGGACTGTGGTCATGAG). For detection, a standard SYBR green protocol as recommended by the manufacturer was used on a LightCycler 480 system (Roche).

### Software and statistics.

We used Microsoft Word and Excel. GraphPad Prism 6.0 to 8.0 was used for statistical analyses and to generate graphs. The statistical tests used are indicated in the figure legends. All figures were generated with CorelDrawX7. SICM images were acquired and processed with Igor Pro 6.37 and MFP-3D software v.10 (Asylum Research). Other software used included MACS Quantify (Miltenyi), Flowlogic (Inivai) for flow cytometry, and LightCycler v.4.1 (Roche). Gene ontology analysis was done with Enrichr (https://amp.pharm.mssm.edu/Enrichr/) (75).
