# Supplementary figures and images for: Comprehensive Analysis of Human Cytomegalovirus- and HIV-Mediated Plasma Membrane Remodeling in Macrophages
Source: mBio. 2021 Aug 17;12(4):e01770-21. doi: 10.1128/mBio.01770-21 (PMC8406226; doi:10.1128/mBio.01770-21)

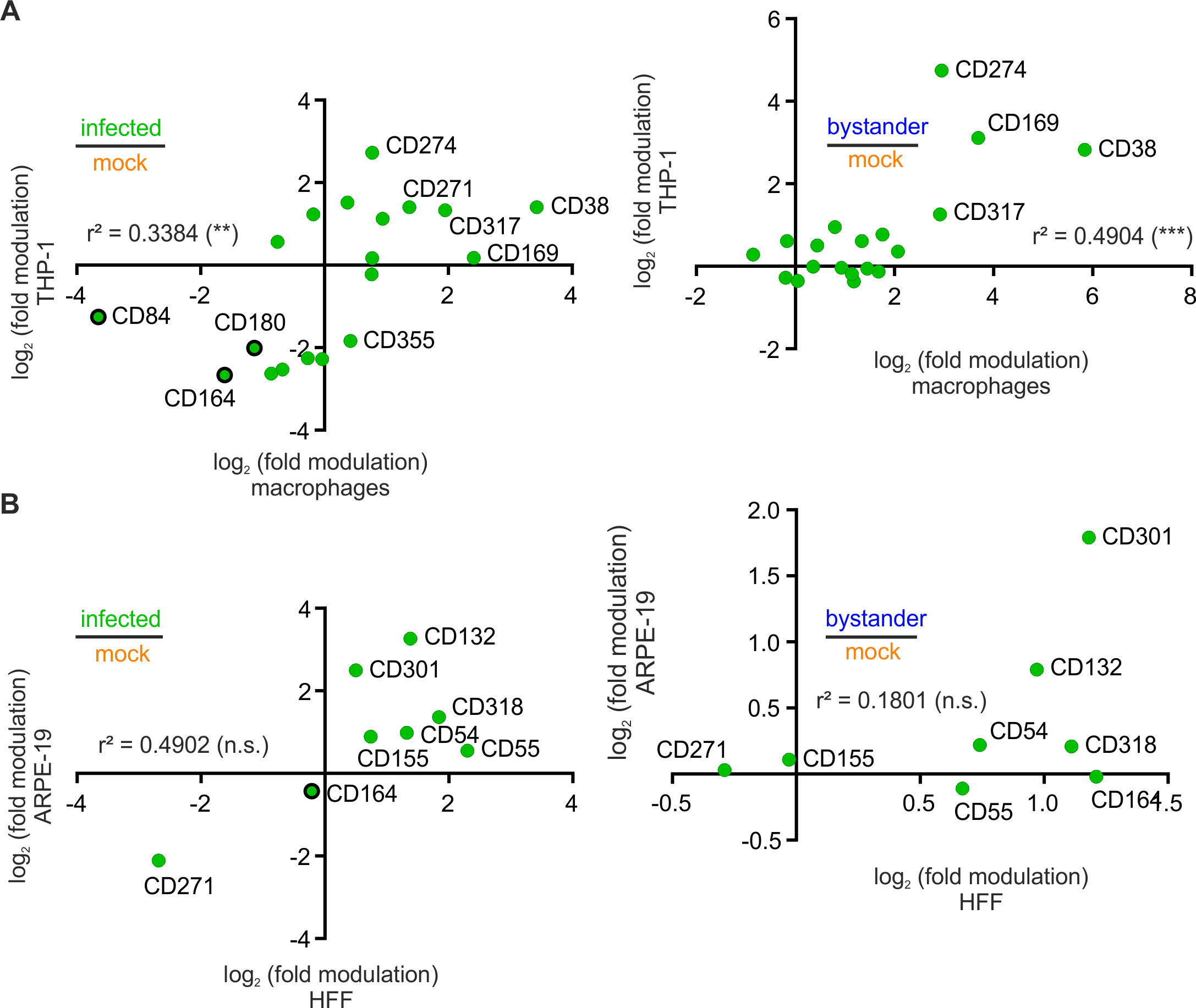

Supplement: FIG S1 [file mbio.01770-21-sf001.tif]

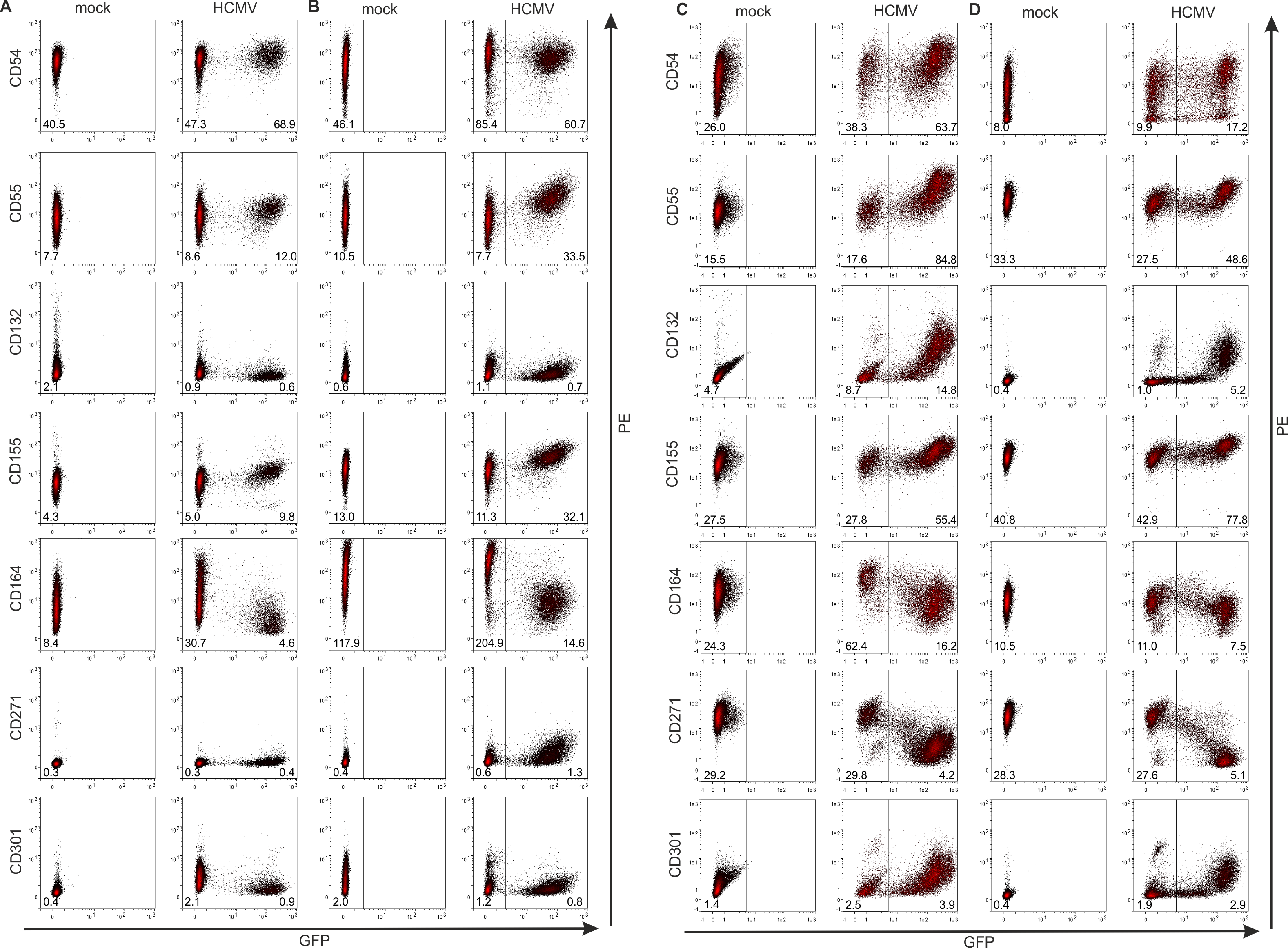

Supplement: FIG S2 [file mbio.01770-21-sf002.tif]

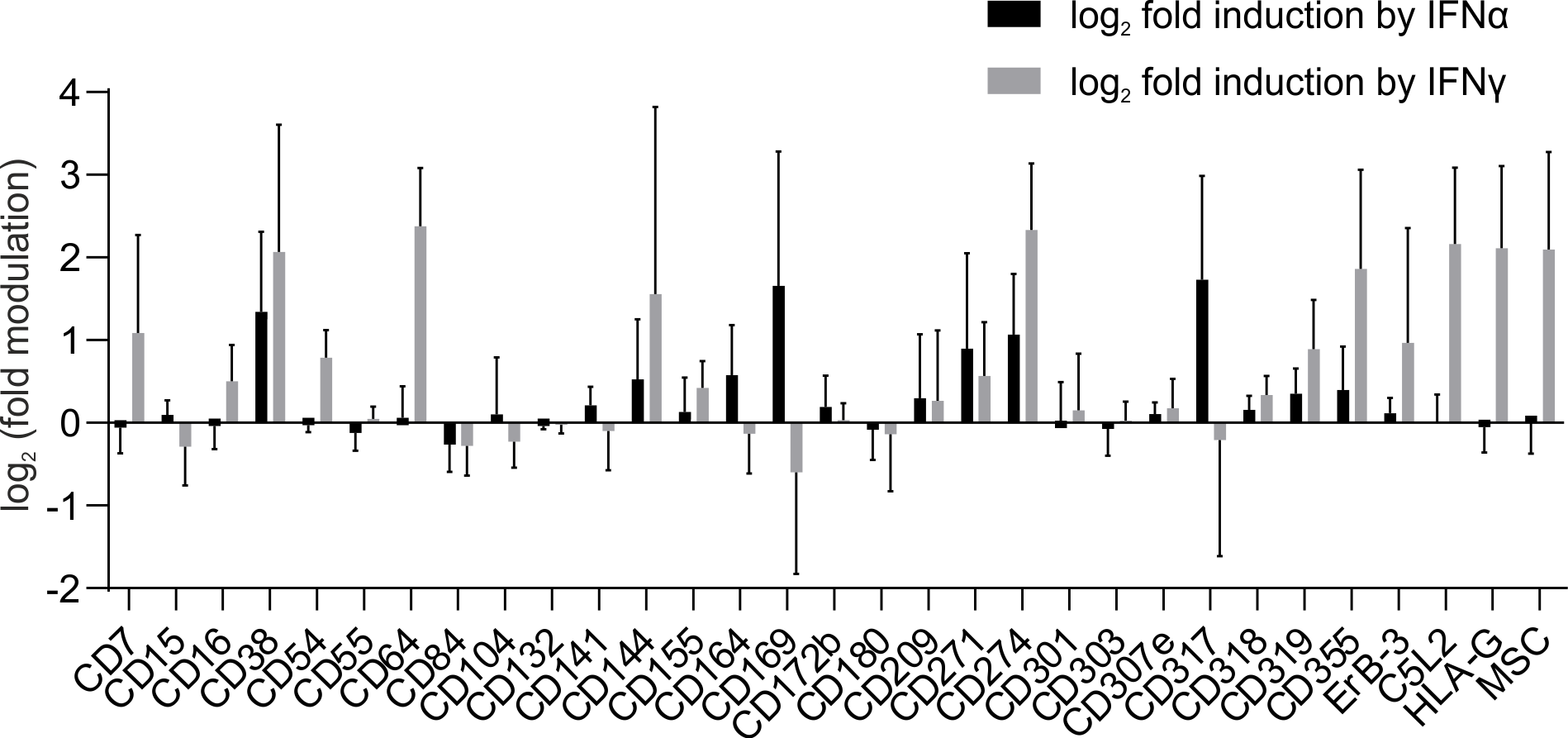

Supplement: FIG S3 [file mbio.01770-21-sf003.tif]

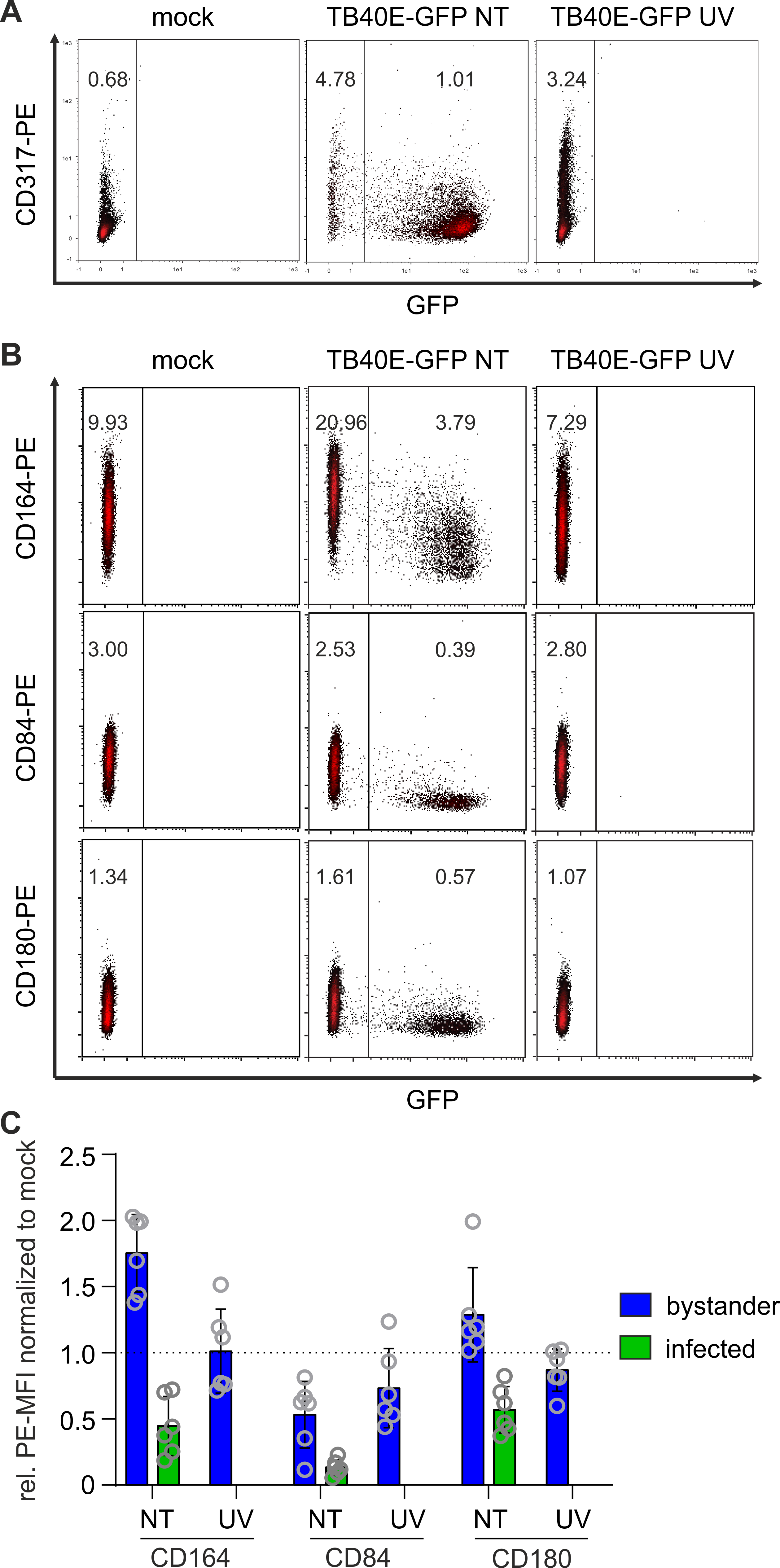

Supplement: FIG S4 [file mbio.01770-21-sf004.tif]
